# Cardiac magnetic resonance in the maze of myeloma-related cardiovascular complications: a decisive diagnostic role

**DOI:** 10.1093/ehjcr/ytag005

**Published:** 2026-01-08

**Authors:** Annagrazia Cecere, Michele Pio Vallario, Martina Perazzolo Marra

**Affiliations:** Department of Cardiac, Thoracic, and Vascular Sciences and Public Health, University of Padua, Via Giustiniani 2, Padua 35128, Italy; Department of Medical and Surgical Sciences, University of Foggia, Foggia 71122, Italy; Department of Cardiac, Thoracic, and Vascular Sciences and Public Health, University of Padua, Via Giustiniani 2, Padua 35128, Italy

**Keywords:** Cardiac magnetic resonance, Multiple myeloma, Myocarditis, Cardiac amyloidosis, Left ventricular hypertrophy

**This editorial refers to ‘Challenging features of left ventricular wall thickening in a young patient with multiple myeloma and shock: when magnetic resonance imaging makes the difference—a case report’, by C. Galdieri *et al*., https://doi.org/10.1093/ehjcr/ytaf590.**

Multiple myeloma (MM) remains a major challenge in contemporary cardio-oncology. Although it primarily affects older adults, cases in younger individuals introduce an additional level of clinical complexity.^1^ The disease course is frequently intertwined with cardiovascular complications, more common and clinically relevant than initially recognized, arising from the underlying plasma cell disorder, the cardiotoxicity of therapeutic regimens, or infectious complications in immunosuppressed phases.^1^ Despite their substantial prognostic impact, these cardiovascular manifestations may progress silently until advanced stages, making timely and accurate differential diagnosis essential for guiding effective management. This need is particularly evident in patients undergoing intensive treatment strategies such as high-dose chemotherapy and autologous stem cell transplantation, where the cardiovascular system is especially vulnerable.^2^ In this context, an early detection of cardiac involvement and a prompt differentiation among potential causes are therefore critical, as delays in diagnosis can rapidly worsen outcomes in this already fragile population.^3^

In this setting, cardiac magnetic resonance (CMR) emerges as an exceptionally powerful diagnostic tool. Beyond its well-established value in assessing cardiac morphology and function, CMR offers a unique ability to characterize myocardial tissue with remarkable accuracy.^4^ The combined evaluation of macroscopic fibrosis through late gadolinium enhancement (LGE) and interstitial fibrosis via native or post-contrast T1 and T2 mapping enables a nuanced distinction among various phenotypes of ischaemic and non-ischaemic cardiomyopathies.^5–7^ This capability is particularly crucial when echocardiographic findings are ambiguous, a scenario frequently encountered in MM patients due to suboptimal acoustic windows, treatment-related fluid retention, or the intrinsic myocardial alterations of infiltrative disorders.^1,8^ So, in MM patients, the ability of CMR to identify specific left ventricular (LV) fibrosis patterns becomes diagnostically transformative.

The case described by Galdieri *et al*.^9^ illustrates the complex management of a young patient with MM, who developed an acute cardiovascular complication. A 44-year-old man, in the aplastic phase following an autologous stem cell transplantation, presented with cardiogenic shock and marked LV wall thickening on echocardiography. The initial differential diagnosis appropriately considered immunoglobulin light-chain (AL) amyloidosis, an infiltrative cardiomyopathy associated with rapid haemodynamic decline and high mortality, as well as acute myocarditis, a known complication in immunosuppressed patients, including those experiencing cytomegalovirus (CMV) reactivation. Because the echocardiographic findings were non-specific, CMR was promptly performed to achieve a more accurate myocardial tissue characterization and to guide targeted treatment. Cardiac magnetic resonance confirmed severe biventricular dysfunction and revealed diffuse myocardial oedema with increased extracellular volume (ECV), consistent with an active inflammation. Late gadolinium enhancement was predominantly localized to the subepicardial regions of the basal and mid-lateral LV wall, a pattern that strongly favours acute myocarditis over infiltrative disease.

This diagnostic distinction becomes even more meaningful in light of recent evidence regarding the presence of myocardial oedema in cardiac amyloidosis. Recent evidence in fact has shown that myocardial oedema is not uncommon in cardiac amyloidosis. Kotecha *et al*.^10^ demonstrated that patients with untreated AL amyloidosis exhibit significantly higher T2-mapping values compared with those with transthyretin amyloidosis (ATTR) and healthy controls, suggesting the presence of active myocardial oedema, a finding associated with worse prognosis. Similarly, Ridouani *et al*.^11^ confirmed the ability of T2 mapping to distinguish AL from ATTR amyloidosis, although ECV emerged as the strongest predictor of clinical outcomes. Taken together, these observations indicate that, even though both conditions may display myocardial oedema, the pattern and distribution of LGE remain essential for establishing a reliable differential diagnosis. In fact, myocarditis is characterized by patchy, non-ischaemic LGE predominantly affecting the lateral or inferolateral wall (*Figure 1A–E*); conversely, in AL amyloidosis, LGE typically involves diffuse subendocardial or transmural enhancement with abnormal gadolinium kinetics^12–14^ (*Figure 1F–J*). Therefore, the LGE distribution on CMR, combined with the patient’s immunosuppression following transplantation and the documented CMV viremia, strongly supported a diagnosis of fulminant CMV-related myocarditis presenting with cardiogenic shock. In this case, the contribution of CMR is two-fold. First, even though endomyocardial biopsy remains the gold standard for the final diagnosis, it is recommended in high-risk myocarditis, and/or persistent haemodynamic instability, and/or selected intermediate-risk scenarios (Class I C); however, in this specific setting, it was contraindicated because of the patient’s thrombocytopenia.^15^ Second, by providing a non-invasive diagnosis, CMR enabled the prompt initiation of antiviral therapy and broad-spectrum antimicrobial coverage, leading to a rapid improvement in cardiac function and ultimately a complete recovery.^15^

**Figure 1:**
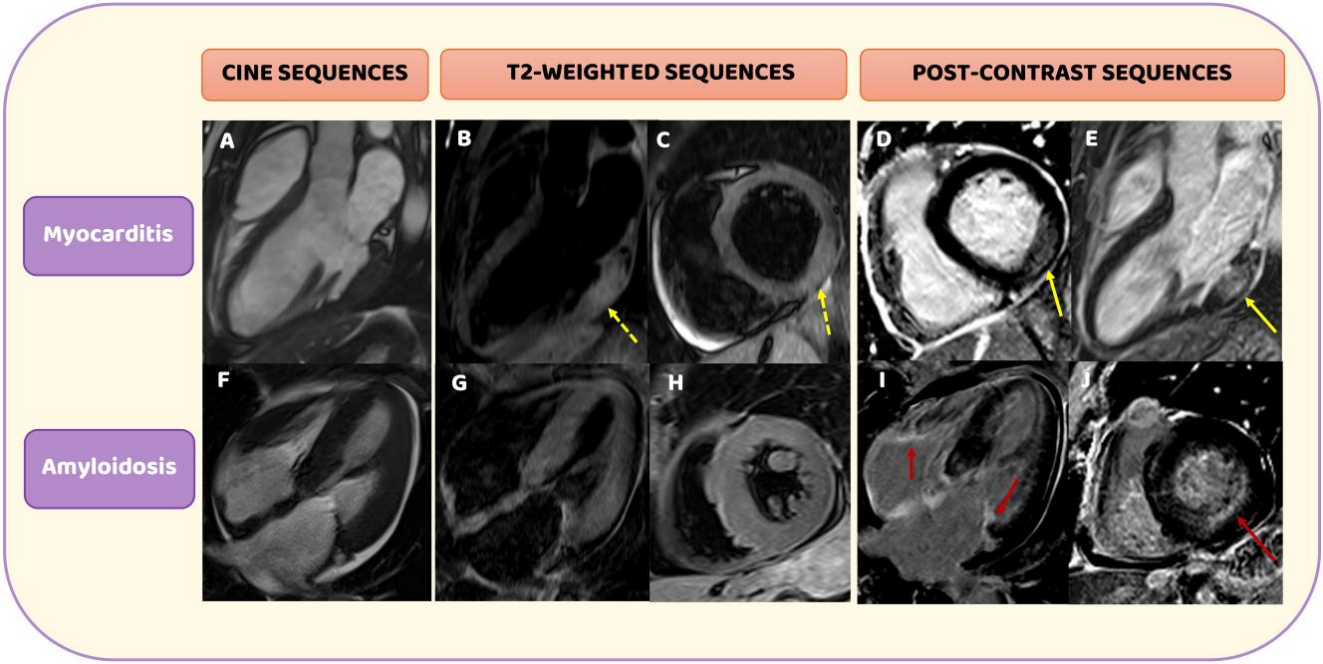
Cardiac magnetic resonance cine, T2-weighted, and post-contrast sequences in patients with myocarditis and cardiac amyloidosis. (*A–E*) A representative case of a 31-year-old male patient with myocarditis and left ventricular hypertrophy (*A*), demonstrating myocardial oedema on T2-weighted images (*B–C*, dotted yellow arrows) and subepicardial late gadolinium enhancement in the basal inferolateral wall on post-contrast sequences (*D–E*, yellow arrows). (*F–J*) A representative case of a 72-year-old female patient with cardiac amyloidosis and concentric wall thickening (*F*). No myocardial oedema is observed on T2-weighted sequences (*G–H*). Post-contrast images reveal diffuse subendocardial late gadolinium enhancement involving the basal inferolateral wall (*J*, red arrow) and extending to the atrioventricular valves and atrial walls (*I*, red arrows).

In summary, this case underscores essential considerations in the management of cardiovascular complications in MM. First, patients undergoing intensive treatments, particularly autologous stem cell transplantation, are exposed to multiple factors capable of precipitating acute cardiac dysfunction, including immunosuppression, treatment toxicity, inflammatory activation, and infectious triggers. Because infiltrative, inflammatory, and stress-related cardiomyopathies may share overlapping phenotypes, a rigorous differential diagnosis is crucial. Second, the case reinforces the central diagnostic work-up of CMR in patients with unexplained LV ventricular hypertrophy or inconclusive echocardiography. Its ability to non-invasively characterize myocardial tissue makes it a cornerstone in differentiating cardiomyopathies with hypertrophic phenotypes.^16^ This approach is fully aligned with the forthcoming 2025 ESC Guidelines on Myocardial and Pericardial Diseases, which emphasize CMR as the advanced imaging modality of choice for suspected myocarditis, particularly in unstable patients or when diagnostic uncertainty persists (Class I B).^15^

Overall, the case report by Galdieri *et al*. illustrates how, in high-risk cardio-oncologic scenarios, timely and precise imaging is integral to prognosis. When MM patients present with cardiogenic shock and increased LV wall thickness, rapid differentiation among potential aetiologies is imperative, as management strategies and outcomes differ profoundly. Although positioned as a second-line examination after echocardiography, CMR frequently becomes the decisive diagnostic step that resolves ambiguity and directs appropriate, aetiology-specific therapy.

## Lead author biography


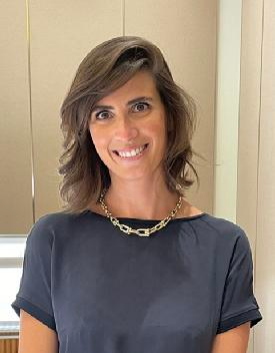


Annagrazia Cecere, MD, PhD student, is an Assistant Professor of Cardiology at the University of Padua. Her research focuses on cardiovascular magnetic resonance, arrhythmic mitral valve prolapse, and rare arrhythmic cardiomyopathies. She has published more than 50 indexed papers (h-index 12) and delivered over 60 invited lectures. Dr Cecere holds ESC Level III Certification in Cardiovascular Magnetic Resonance and serves on the editorial boards of several indexed peer-reviewed journals. As an educator, she teaches core cardiology courses to medical students and supervises residents and post-graduate fellows during clinical and research rotations. Finally, she is a lecturer in a second-level Master's programme in cardiac magnetic resonance and rare arrhythmic cardiomyopathies at the University of Padua.

## Data Availability

All data are included in the publication.
